# Update on Viral Infections Involving the Central Nervous System in Pediatric Patients

**DOI:** 10.3390/children8090782

**Published:** 2021-09-06

**Authors:** Giovanni Autore, Luca Bernardi, Serafina Perrone, Susanna Esposito

**Affiliations:** 1 Pediatric Clinic, Pietro Barilla Children’s Hospital, Department of Medicine and Surgery, University of Parma, Via Gramsci 14, 43126 Parma, Italy; giovanniautore@gmail.com (G.A.); Bernardi.luca91@gmail.com (L.B.); 2 Neonatology Unit, Pietro Barilla Children’s Hospital, Department of Medicine and Surgery, University of Parma, Via Gramsci 14, 43126 Parma, Italy; serafina.perrone@unipr.it

**Keywords:** central nervous system (CNS) infection, encephalitis, meningitis, pediatric infectious disease, viral infection

## Abstract

Infections of the central nervous system (CNS) are mainly caused by viruses, and these infections can be life-threatening in pediatric patients. Although the prognosis of CNS infections is often favorable, mortality and long-term sequelae can occur. The aims of this narrative review were to describe the specific microbiological and clinical features of the most frequent pathogens and to provide an update on the diagnostic approaches and treatment strategies for viral CNS infections in children. A literature analysis showed that the most common pathogens worldwide are enteroviruses, arboviruses, parechoviruses, and herpesviruses, with variable prevalence rates in different countries. Lumbar puncture (LP) should be performed as soon as possible when CNS infection is suspected, and cerebrospinal fluid (CSF) samples should always be sent for polymerase chain reaction (PCR) analysis. Due to the lack of specific therapies, the management of viral CNS infections is mainly based on supportive care, and empiric treatment against herpes simplex virus (HSV) infection should be started as soon as possible. Some researchers have questioned the role of acyclovir as an empiric antiviral in older children due to the low incidence of HSV infection in this population and observed that HSV encephalitis may be clinically recognizable beyond neonatal age. However, the real benefit-risk ratio of selective approaches is unclear, and further studies are needed to define appropriate indications for empiric acyclovir. Research is needed to find specific therapies for emerging pathogens. Moreover, the appropriate timing of monitoring neurological development, performing neuroimaging evaluations and investigating the effectiveness of rehabilitation during follow-up should be evaluated with long-term studies.

## 1. Introduction

Infections of the central nervous system (CNS) are mainly caused by viruses, and these infections can be life-threatening in pediatric patients [1,2,3]. The incidence rate of CNS viral infections ranges from 10 to 20 cases per 100,000 children per year in high-income countries [4,5]. The actual incidence in many low-income countries in the pediatric population is still unclear, and regional reports are often affected by recurring outbreaks. The global epidemiology of CNS viral infections has been influenced by the spread of more effective diagnostic techniques, the introduction of vaccination programs, and the emergence of new pathogens. In a large observational study conducted in the UK over 50 years, the mean admission rate for viral meningitis was 13.5 per 100,000 children per year [6]. The authors observed an important decrease in incidence in the late 1980s for children aged 1–14 years, probably due to the introduction of immunization against mumps, which was a leading cause of CNS infection. However, an increase in admission rates since 2003 has been reported, coinciding with increased use of PCR on cerebrospinal fluid samples. In East Asian countries, similar trends have been reported after immunization programs against Japanese encephalitis virus (JEV), which has been the regional leading reported cause of acute encephalitis in children [7,8]. Two peaks of incidence can be identified: the first peak in infants aged under 1 year and a second peak in children approximately five years old [9,10]. Most cases arise during summer and autumn because enteroviruses and arboviruses have major activity during these periods. Viral CNS infections often affect immunocompetent children; in fact, in their large study, Hasburn et al. observed that 91.4% of children with viral meningitis or encephalitis had no reported underlying conditions [5].

Although prognosis is often favorable, mortality and long-term sequelae due to viral meningitis and encephalitis can occur [3]. Moreover, the burden for public health systems is high for these conditions, with a mean associated hospitalization cost of $60,000 per patient [11]. However, viral meningitis and encephalitis represent a diagnostic challenge, and specific treatment options are still scarce. The aims of this narrative review were to describe the specific microbiological and clinical features of the most frequent pathogens and to provide an update on the diagnostic approaches and treatment strategies for viral CNS infections in children.

We conducted a review of recent literature by examining the Medline (Medical Literature Analysis and Retrieval System Online) database using the search engines PubMed and Google Scholar. The keywords used were “viral/aseptic meningitis”, “viral/aseptic encephalitis”, “p(a)ediatric”, and “children”. We included clinical trials, observational studies, reviews, and meta-analyses on acute viral infections affecting the CNS. The exclusion criteria were patients older than 18 years, nonviral infections, case series with fewer than 20 patients, and articles not published in English.

## 2. Epidemiology

### 2.1. Enteroviruses

Enteroviruses (EVs) represent the main viral pathogens causing meningitis in children, accounting for 85% of meningitis cases. The serotypes of circulating nonpolio-EVs change every year, but some of these EVs maintain an important prevalence over time [12]. Between 2014 and 2016, the most prevalent serotypes identified in the United States were coxsackieviruses A6 and B3 and echoviruses 30, 18, 9, and 11. The epidemic in the United States during 2014 aroused concern about EV D68, which was later reported in some other outbreaks in Europe [13,14]. EV71 is emerging as an important cause of encephalitis, meningitis, and myelitis [15].

EVs have predominantly fecal-oral transmission, and humans are the only reservoir of infection [12,13,14,15]. In very rare cases, EV infection can occur through the inhalation of droplets. The incubation period of EV infection ranges between three and six days. The pathogenesis of EV infection is unclear. Specific receptors on enterocytes are probably bound by viral particles that reproduce themselves in the Peyer’s patches of the lamina propria [16,17,18]. Other viral elements across the nasopharynx reach the lymphatics of the respiratory tract. The first viremic phase carries EVs to the spleen, liver and heart, and in these organs, the virus rapidly replicates, leading to a second viremic phase with signs and symptoms of infection. The CNS may be involved during both phases [16,17,18].

### 2.2. Human Parechoviruses

Human parechoviruses (HPeVs) are members of the family *Picornaviridae* and are widespread worldwide. HPeVs are transmitted by the fecal-oral route, although HPeVs can also reproduce in the respiratory tract [19]. The most common serotypes are HPeV1, followed by HPeV3, HPeV4, and HPeV6 [20].

In the pediatric population, HPeV1, HPeV2 and HPeV4–8 mainly cause mild gastrointestinal or respiratory illness, but occasionally more serious diseases have been reported, including myocarditis, encephalitis, pneumonia, meningitis, flaccid paralysis, Reye’s syndrome and fatal neonatal infection [21,22,23]. Harvala et al. reported that HPeV3 infection is more frequent than HPeV1 infection in younger children (i.e., neonates and infants under 3 months), in whom it can cause severe diseases such as sepsis, meningitis and encephalitis [19,24].

### 2.3. Herpesviruses

Infections caused by herpes simplex viruses (HSVs) occur worldwide every season, with humans as the only reservoir [25]. After the first HSV infection, the reactivation of HSV is possible, especially in immunocompromised subjects. HSV-1 accounts for 25% of neonatal infections due to HSV, and, particularly in newborns, CNS infections may cause serious sequelae. In fact, HSV-1 is commonly the cause of sporadic necrotizing encephalitis, accounting for approximately 85% of all cases [16]. Despite concern due to its potential severity in young infants, the actual incidence of HSV meningoencephalitis seems lower than that expected. Cruz et al. performed a retrospective study among infants aged under 3 months who presented with suspected CNS infections in 23 emergency departments of the United States, reporting a prevalence of HSV infections of approximately 0.4% [26]. Moreover, the incidence of encephalitis caused by HSV among the pediatric general population is approximately 1/1,000,000 children and adolescents per year [27]. HSV-2 causes 75% of HSV neonatal infections. Vertical transmission is more common during maternal primary infection, when the rate of transmission is approximately 40% versus 3% during recurrent infection [28,29]. HSV-2 is particularly associated with Mollaret’s meningitis, characterized by repeated episodes of meningitis lasting 2 to 5 days occurring weeks to years apart. Epstein-Barr virus (EBV) has also been associated with this pattern [30].

Regarding varicella zoster virus (VZV), the incidence of VZV infection in children was reduced due to the introduction of a vaccination. In approximately 1% of varicella cases, CNS complications may occur, often represented by cerebellar ataxia and encephalitis [31]. In rare conditions known as zoster sine herpete, an analysis of cerebrospinal fluid (CSF) may show pleocytosis and the presence of viral DNA, but the typical chicken pox skin lesions may not be present [32].

Meningitis is an uncommon complication of Epstein-Barr virus (EBV) disease, and it mostly occurs during primary infection [3].

Acquired CNS infection caused by cytomegalovirus (CMV) is a rare event in healthy children [3]. However, it may be a severe complication in the immunocompromised population associated with high rates of morbidity and mortality.

The actual incidence of CNS infections caused by herpes human virus 6 and 7 (HHV-6 and HHV-7, respectively) is unclear, but aseptic meningitis is considered a rare complication in healthy children that may occur with or without other symptoms, such as febrile seizures and exanthema subitum [33,34]. The outcome is typically good.

### 2.4. Arboviruses

In this group of arthropod-borne RNA viruses, three families relevant to human CNS infections are included: *Togaviridae, Flaviviridae* and *Bunyaviridae*.

The Arboviruses endemic in the United States are West Nile virus (WNV), eastern equine encephalitis virus, western equine encephalitis virus, La Cross virus, and St. Louis encephalitis virus. The most common arboviral causes of meningitis in children were WNV and La Crosse virus [35,36]. Mosquitoes are the insect vector, and through their bite, Arboviruses are inoculated subcutaneously; after replication in muscles and skin, the virus spreads to the reticuloendothelial system or to the CNS. The average incubation period is approximately 15 days. In the United States, Arboviruses are the second main cause of viral meningitis after EVs [37]. Most infected children are asymptomatic; approximately 25% report fever or headache, but 1% may develop meningitis [38,39]. WNV is still a serious public health problem in Europe, and hundreds of cases of WNV infection have been reported in recent years from June to August [40]. In Italy, West Nile virus lineage 2 (WNV-2) was isolated in humans, and the associated vector mosquitoes probably diffused from Eastern European countries [41]. In the Emilia-Romagna region of Italy, from 2013 to 2017, WNV-2 caused sporadic human infections, with from 7 to 20 cases per year reported [42,43,44].

Other Arboviruses endemic in Europe are those that cause tick-borne encephalitis. Tick-borne encephalitis is caused by three closely related virus members of the Flaviviridae family, known as the Russian spring-summer encephalitis subtype (or eastern subtype), Siberian subtype (or Vasilchenko virus), and Central European encephalitis subtype (or western subtype). The main vectors are ticks of the genus *Ixodes* [45].

### 2.5. Influenza Viruses

Neurological complications are reported in up to 15% of children with influenza [46,47]. Moreover, influenza viruses account for 2–11% of encephalitis cases in childhood. Neurological disease associated with influenza virus infection is often defined as influenza-associated encephalitis/encephalopathy (IAE). The term encephalopathy is preferred because influenza virus is rarely neuroinvasive [46,47]. Direct CNS invasion by influenza virus is rare, although viral RNA in CSF has been detected in a few cases [48].

Influenza may cause a wide spectrum of neurological signs/symptoms, from a mildly altered mental state, vertigo, febrile seizures, and demyelinating disease to status epilepticus, meningitis, and stroke [49]. Mastrolia et al., in a retrospective study, underlined that all children presenting acute neurological features during the influenza season should be evaluated for influenza-associated CNS complications even if respiratory involvement is mild [46]. Furthermore, the absence of underlying diseases or other risk factors is not protective against influenza-associated neurological complications [46].

### 2.6. Lymphocytic Choriomeningitis Virus

Lymphocytic choriomeningitis virus (LCMV) is a rare cause of human CNS infections in developed countries. The risk of contracting LCMV infections is related to contact with secretions of rodents, especially during the winter months [50].

### 2.7. Severe Acute Respiratory Syndrome Coronavirus 2 (SARS-CoV-2)

Studies about the neuroinvasive potential of SARS-CoV-2 are encouraged by reports of neurological manifestations in 36% of adult patients [51]. A systematic review and meta-analyses of studies involving approximately 3700 pediatric COVID-19 patients showed that 17% of them had nonspecific neurological conditions and 1% had signs and symptoms of severe brain involvement [52]. In patients with multisystem inflammatory syndrome in children (MIS-C), the incidence of abnormal neurological findings has been reported in up to 55% of cases [53]. Fortunately, in most children, neurological manifestations, mainly represented by headache and anosmia, are mild, transient and do not significantly complicate the COVID-19 course. However, in some children with SARS-CoV-2 infection, very severe clinical problems associated with relevant alterations of neuroimaging, electroencephalography, nerve conduction studies and electromyography findings can develop.

Several mechanisms have been postulated to explain the acute and postacute neurological manifestations of COVID-19. Although neurological damage can simply be the consequence of hypoxia, hypotension and liver and renal insufficiency, which are characteristic of the most severe cases of COVID-19, a number of specific mechanisms are considered the main causes of CNS and PNS damage [54,55]. SARS-CoV-2 may infect olfactory neurons and subsequently invade the brainstem, basal ganglia and cortex, with a direct destructive effect [54]. Moreover, as SARS-CoV-2 depletes angiotensin-converting enzyme 2 (ACE-2), causing significant renin-angiotensin system disequilibrium, it is thought that following infection, a prothrombotic state with impaired large vessel and microvascular blood flow can occur and lead to an increased risk of thrombotic and hemorrhagic stroke [54]. The most important mechanism seems, however, to be immune dysregulation leading to autoimmunity and hyperinflammation [54]. Moreover, in some patients with acute disseminated encephalomyelitis (ADEM)-like magnetic resonance images, antibodies against brain components have been detected, strongly suggesting that COVID-19 can be associated with an immune-mediated pathology [56]. However, regardless of the pathophysiology, in all these cases, early identification, prompt diagnosis and careful implementation of the most effective therapeutic interventions are needed to ensure the resolution of the acute disease and the potential long-term abnormalities.

## 3. Diagnosis

In almost all cases, clinical features cannot differentiate viral from bacterial CNS infections, and often the CSF profiles of bacterial and viral infections overlap. For this reason, pending the results of CSF cultures and virological studies, empiric treatment should cover the most common and probable pathogens [57].

An accurate history and physical examination may provide hints for the diagnostic approach. Classic symptoms and signs of meningeal inflammation (e.g., fever, stiff neck, headache, photophobia, Kernig and Brudzinski signs) associated with typical findings of viral infections (e.g., rash, specific skin lesions, herpangina, conjunctivitis, pharyngitis, vomiting, diarrhea) suggest a viral etiology [57]. Focal motor or sensory deficits suggest encephalitis that is often caused by viruses [57]. Moreover, exposure to ill contacts, ticks, mosquitoes, or rodents and seasonal outbreaks of viral infections should be considered [57].

Lumbar puncture (LP) in pediatric patients should be performed as soon as possible when CNS infection is suspected [58]. Indications for neuroimaging before LP are limited to severely depressed mental status, a history of hydrocephalus, recent trauma or neurosurgery with a CSF shunt, papilledema, and focal neurological deficits other than cranial nerve VI or VII palsy [57,58]. However, in these conditions, blood cultures and empiric antibiotics should not be delayed before neuroimaging. Conditions representing potential contraindications for LP are limited to the risk for cerebral herniation (i.e., intracranial space-occupying lesions with mass effects, abnormal intracranial pressure, and Arnold-Chiari malformations), increased bleeding risk, and local infections at the puncture site [56].

Table 1 summarizes laboratory, microbiological, and imaging evaluations for suspected CNS infections. Children are likely to have viral meningitis when lumbar puncture reveals a CSF white blood cell count <500/µL with >50% mononuclear cells, CSF protein <100 mg/dL and normal CSF glucose [58]. However, CSF and blood samples should always be collected and sent for Gram staining and bacterial culture when CNS infection is suspected. The Bacterial Meningitis Score (BMS) is a validated tool to assess the risk of bacterial meningitis in pediatric patients and can be used in differential diagnoses [59]. The BMS includes the following variables: a positive CSF Gram stain (2 points), a CSF absolute neutrophil count ≥1000 cells/µL (1 point), CSF protein ≥80 mg/dL (1 point), a peripheral blood neutrophil count ≥10,000 cells/µL (1 point), and a history of seizure before or at the time of presentation (1 point). A score of 0 indicates a “very low risk” of bacterial meningitis. In a meta-analysis of eight validation studies, a score ≥1 predicted bacterial meningitis with a sensitivity and specificity of 99.3% and 62.1%, respectively. However, the most useful feature of the BMS is its negative predictive value of 99.6% [60].

In cases of suspected viral CNS infection, CSF samples should be sent for analysis by polymerase chain reaction (PCR) to detect viral nucleic acids. PCR has largely replaced viral culture and serological diagnosis of viral meningitis, improving diagnostic accuracy. Moreover, PCR is highly sensitive and specific, and results are available sooner than viral culture [61,62,63,64]. PCR for EVs and HSV should always be performed, and whether other pathogens may be tested depends on epidemiology and clinical findings. To improve viral detection, stool and throat swabs can also be sent for PCR or viral culture [62]. Serologies may be obtained for the less common viruses; moreover, the presence of specific IgG and IgM for WNV in CSF may also be tested [62]. When HSV infection is suspected, cultures of skin lesion samples, if present, and PCR on blood samples should be performed [62].

Pediatric acute-onset neuropsychiatric syndrome (PANS), i.e., a newly defined symptom-based condition that mainly occurs in children and adolescents, have described after viral infections, including SARS-CoV-2 [65,66]. It is an emerging immune-mediated encephalopathy characterized by sudden onset of seemingly inexplicable complex neuropsychiatric symptoms, including obsessions, compulsions, and heterogeneous tics [65]. PANS has a relapsing–remitting symptom course, and further research into its diagnosis and management is warranted.

## 4. Treatment

Pediatric patients with symptoms and signs of CNS infections always require hospitalization to receive supportive care and specific treatment [57]. Patients should be treated in an isolation room with adequate oxygenation, ventilation, and circulation, and acetaminophen for headache, pain, and fever should be provided. Some patients, especially those in whom HSV-1 and HSV-2 are involved, may require intensive supportive care. Intravenous fluid therapy is an important aspect of supportive care. The cornerstone of supportive care is the balance between treating hypovolemia and avoiding inappropriate secretion of antidiuretic hormone syndrome (SIADH) [57]. This frightening complication occurs with oliguria, serum sodium < 135 mEq/L, serum osmolality < 280 mOsm/L, and urine osmolality >100 mOsm/L. If this situation occurs, fluid restriction (<1200 mL/m^2^/day) is indicated. There is no evidence on the benefits of corticosteroids in viral CNS infections, regardless of their etiology.

When viral infection of the CNS is suspected, empiric antiviral therapy with acyclovir should be administered until the results of specific PCR for HSV on CSF samples are found to be negative [67,68,69,70,71,72]. HSV encephalitis is a devastating infection that may have a subtle presentation and needs prompt therapy [73]. In newborns, HSV should also be ruled out with skin culture when skin lesions occur or blood PCR before empiric acyclovir is discontinued [74,75].

Driven by this scenario, the use of empiric acyclovir has increased in recent decades, raising concern about its appropriateness considering that an empiric antiviral course, particularly beyond neonatal age, may be associated with adverse effects and may prolong hospitalization [74,76]. On the other hand, the incidence of HSV encephalitis seems very low in children and adolescents, accounting for 1 case every 1,000,000 children and adolescents per year and representing 0.4% of encephalitis cases in infants aged less than 3 months [67]. Some recent studies have suggested that empiric treatment in older infants and children should be considered only in appropriate clinical scenarios in which the patient has clinical signs of acute encephalitis (i.e., focal neurological impairment, seizures, altered mental status, suggestive findings in neuroimaging or electroencephalography) and in immunocompromised patients [76,77]. However, this approach is often unfeasible due to the frequent overlap of clinical presentations in children with viral meningitis or viral encephalitis. Moreover, the results from molecular analysis of CSF, including PCR for HSV, are currently quickly available, allowing the discontinuation of acyclovir after a few doses when not necessary and minimizing the impact of acyclovir use on the hospital length of stay and costs. Regarding the potential nephrotoxicity of acyclovir, recent studies reported that adverse effects can be common but are usually not severe, and the risk for acute kidney injury is increased in sicker patients who need intensive care, in patients with confirmed HSV infections, and in patients receiving concomitant nephrotoxic drugs, suggesting that most events may be related to the underlying clinical conditions [78,79,80,81]. To date, empiric acyclovir treatment in patients with suspected viral CNS infection remains recommended until HSV infection is excluded. Table 2 summarizes the recommended acyclovir regimen.

Acyclovir should also be administered in children and adolescents with signs of VZV infection, particularly in children with CSF pleocytosis and mucocutaneous vesicles, seizures, lethargy, thrombocytopenia, hepatomegaly, ascites, or elevated transaminases [82]. The development of meningitis due to VZV reactivation is relatively uncommon, particularly in immunocompetent patients. Only a few cases have been described in the literature in pediatric patients. In these cases, prompt therapy with acyclovir is recommended [83].

Other selected antiviral agents have been tested against some viral pathogens, but there is little evidence for effective specific therapies.

Recent studies have investigated the activity of capsid inhibitors against EVs, which impair viral attachment and uncoating [84]. In a trial of 61 neonates with suspected CNS infection due to EVs, who were randomly assigned to seven days of pleconaril or placebo, there was a trend toward more rapid viral clearance and lower overall mortality among pleconaril-treated infants [85]. Another clinical trial on EV meningitis showed only a modest benefit with pleconaril [86]. However, pleconaril is not yet available in the market.

Antiviral therapy is not usually indicated for acquired CMV infection in immunocompetent children because this infection is usually self-limited; however, treatment may be considered in immunocompetent children with serious symptomatic infections, and therapy is recommended in immunocompromised patients [87,88]. In these patients, ganciclovir 12 mg/kg/day i.v., divided into two daily doses for 2–3 weeks is the recommended regimen. In immunocompromised patients, a subsequent maintenance regimen with oral valganciclovir 15 mg/kg/dose twice a day for 12 months is recommended [89].

Arboviral infections are usually treated symptomatically [90]. There are few studies evaluating specific experimental therapies against WNV infection. Gnann JW Jr et al. conducted a clinical trial comparing anti-WNV polyclonal human antibodies versus intravenous immunoglobulin and normal saline, showing no clinical benefits with specific polyclonal antibodies [90]. Acyclovir has been used to treat WNV neuroinvasive disease in nonrandomized case series, but no clinical benefit was observed [91].

Children and adolescents affected by influenza with neurological involvement should be treated with antiviral drugs as soon as possible [92]. The first choice is represented by neuroaminidase inhibitors (NAIs), which are effective against both influenza A and B [93]. Oseltamivir is the treatment of choice. Peramivir is another available NAI licensed for use in children aged over 6 months, but evidence for its effectiveness in patients with severe influenza is lacking [94].

Regarding SARS-CoV-2, almost all children with COVID-19 and neurological manifestations reported up until now have made a complete recovery, although in some children, this recovery has occurred after several weeks. Standard COVID-19 treatment is recommended in these patients [95].

## 5. Prognosis

Most children and adolescents with viral CNS infection recover completely in 7 to 10 days without specific treatments. However, the prognosis of children and adolescents with viral CNS infection depends on patient age and the pathogen involved [96].

The mortality rates of children with untreated encephalitis caused by HSV reach 70%, and even in treated pediatric patients, long-term sequelae are common (i.e., 30% of the cases) [73].

The exact morbidity and mortality of meningitis caused by EVs are not known. For newborns, the reported rates of sequelae and mortality are up to 74% and 10%, respectively. The most common fatal complications are hepatic failure for echoviruses and myocarditis for coxsackieviruses [97,98]. Immunocompetent infants and children with EV meningitis generally recover without neurological sequelae. However, EV71 meningitis with systemic involvement has been associated with delayed development and reduced cognitive function [99]. Moreover, long-term follow-up studies on children with human parechovirus CNS infections reported neurological disabilities in approximately one-quarter of young infants, particularly when infection occurs in newborns and early infancy [100].

Mortality associated with WNV neuroinvasive disease in children is approximately 1%, which is significantly lower than the rates reported for the adult population, which range up to 14% [101].

Regarding COVID-19, definitive conclusions about its long-term consequences cannot be drawn due to the low number of case studies and the limited follow-up time. On the other hand, the risk that, at least in some children, some long-term neurological problems may persist cannot be excluded.

No other data on the prognosis of viral CNS infections in pediatric patients are available.

## 6. Prevention

Considering the high rate of HSV vertical transmission, cesarean delivery should be considered before the rupture of the membranes in women with active genital skin lesions due to HSV [28]. Moreover, in women with recurrent genital herpetic infections, oral acyclovir before delivery is suggested by the American College of Obstetricians and Gynecologists [102].

To prevent the spread of EVs, simple hygienic measures, such as hand washing after diaper changing, are very useful [103]. Recently, there have been three inactivated enterovirus A71 vaccines licensed in China, and other types of other vaccines are also under development [104,105].

Vaccines against influenza and some arboviruses (Japanese encephalitis virus and tick-borne encephalitis) are available and appear useful in the prevention of CNS infections due to these pathogens [106,107,108,109].

Finally, the availability of vaccines for COVID-19 for adolescents will permit a reduction in the rate of SARS-CoV-2 infection and its complications in the pediatric population [110,111].

## 7. Conclusions

Due to the lack of specific therapies, the management of viral CNS infections is mainly based on supportive care. Diagnostic LP and molecular analysis of CSF samples should be promptly performed, and empiric treatment against HSV infection should be started as soon as possible. Some researchers have questioned the role of acyclovir as an empiric antiviral in older children due to the low incidence of HSV infection in this population and observed that HSV encephalitis may be clinically recognizable beyond neonatal age, thereby encouraging us to limit its use to patients with suspected HSV infection. However, the real benefit-risk ratio of selective approaches is unclear, and further studies are needed to define appropriate indications for empiric acyclovir.

Few specific therapies are available for CNS infections due to viruses other than HSV. Research is needed to find specific therapies for emerging pathogens. Although prognosis is often favorable, neurological sequelae may occur, and children may need long clinical and radiological follow-up. The appropriate timing of monitoring neurological development, performing neuroimaging evaluations and investigating the effectiveness of rehabilitation during follow-up should be evaluated with long-term studies.

## Figures and Tables

**Table 1:** Laboratory, microbiological and imaging evaluations for suspected central nervous system (CNS) infections.

Laboratory tests	Blood culture Blood gas analysis Complete blood cell count with differential and platelet count C reactive protein and procalcitonin Serum electrolytes, renal and hepatic function markers, glucose PT, INR, and PTT
Lumbar puncture and CSF analysis	Cell count with differential, glucose and protein concentrations Gram stain and bacterial culture PCR for most common viruses Specific IgM and IgG in CSF when WNV is suspected
Other microbiological tests	Serological analysis for suspected viruses PCR and culture from other samples (e.g., cultures from skin swab and PCR on blood for HSV)
Neuroimaging	Indications for imaging before LP: severely depressed mental status history of hydrocephalus recent trauma or neurosurgery with a CSF shunt papilledema focal neurological deficits other than cranial nerve VI or VII palsy

CSF, cerebrospinal fluid; HSV, herpes simplex virus; LP, lumbar puncture; PCR, polymerase chain reaction; WNV, West Nile virus.

**Table 2:** Recommended acyclovir regimen for herpes simplex virus (HSV) central nervous system (CNS) infection in pediatric patients.

Age	EV Dosage	Duration in Patients with Confirmed HSV Infection
<3 months	60 mg/kg/day divided every 8 hours	21 days
3 months–11 years	30–45 mg/kg/day divided every 8 hours	14–21 days
>12 years	30 mg/kg/day divided every 8 hours	14–21 days

## Data Availability

Not applicable in a review article.
